# Cases of Cholera with Remarks

**Published:** 1850-03

**Authors:** Henry Yale Smith

**Affiliations:** Resident Physician to the “Moyamensing Cholera Hospital”


### BUFFALO MEDICAL JOURNAL

AND

### MONTHLY REVIEW.

VOL. 5. MARCH, 1850. NO. 10.

### ORIGINAL COMMUNICATIONS.

*Cases of Cholera with remarks by* Henry Yale Smith, M. D., resident Physician to the "Moyamensing Cholera Hospital." Communicated in a Letter to Prof. James Bryan, Philadelphia, Penn.

To Prof. James Bryan.

*Respected Sir:*—The following cases taken from private, and public practice, I respectfully dedicate to you. Some of them were witnessed by yourself. My gratitude for the many favors received from you, independently of your acknowledged standing in your profession, induce me to trespass on your valuable time in presenting to you the following imperfect cases, and remarks, upon the great epidemic of the age.

Very truly your friend,

H. Y. SMITH,

---

*Case 1st.*—I was called, June 25th, to see Wm. S., æt. 20, (white) residing in Christian St. above Fifth, who was attacked with the premonitory symptoms of cholera about three hours previous to my visit. He was suffering from severe cramps in the abdomen and lower extremities, with excessive purging. The discharges were at first of a greenish yellow color, afterwards they resembled the froth of porter. His whole and continued cry was for water, water; so eager indeed was he, that he twice bit off pieces of the tumbler. The treatment consisted in the exhibition of opium and camphor, two grains every hour, with warm drinks, such as warm teas, brandy and camphor, tincture of ginger, etc. Sinapisms to the epigastrium; hot bricks and bottles were applied to his feet, legs and thighs. In about three hours he was quite relieved. Vomiting in this case was absent.

*Case 2d.*—Patrick K., æt. 40, native of Ireland, residing in Spafford street, above Shippen, was attacked July 1st, with spasms of the voluntary muscles, coldness of the extremeties, vomiting, cholic pains, which had been preceded by diarrhœa for five or six days. Opium and camphor, of each twelve grains, made into six pills, one to be taken every half hour. For the vomiting, two gtts. of creosote, in aromatic mucilage was given. Mustard to the abdomen, with hot application to the feet, and the internal exhibition of warm drinks, were resorted to. In two or three hours diaphoresis had set in (or out), and he was out of danger.

*Case 3d.*—Mrs. B., æt. 37, (white), residing in Catherine, above Seventh street, near the hospital. On making a professional visit to the house, at 1 o'clock, P. M., she remarked to me that she feared the cholera, on account of her proximity to the hospital, the patients being carried through the front yard to the institution. At two o'clock, one hour afterwards, she was taken with the disease which she so much dreaded. A messenger was instantly dispatched for me, but not finding me, my friend Dr. A. attended, and prescribed until I came at 8 o'clock. We agreed to give her half a drachm of tincture of opium with twenty drops of tincture of camphor every half hour, until they had the desired effect. Brandy and capsinum, with warm drinks, were repeatedly administered. Sinipisms to the abdomen, with frictions with dry flour and capsicum were continued until 9 o'clock, when diaphoresis came on, which, however, was cold and clammy. At 11, P. M., she had had one stool during the interval, at which time she voided at least half a gallon of a dark, muddy fluid, perfectly inodorous. Feeling confident that she was fast sinking, additional advice was sought, and Dr. H., of Southwark, called in. In consultation we agreed to try Ayre's plan of treatment, viz: giving one grain of calomel every five minutes, with one drop of tincture of opium. A mixture of chloroform and sulphuric ether was ordered. At 4 o'clock, A. M., I hastily obeyed a summons to see her, and found her so low as to be scarcely able to recognize me. I met Drs. H. and A. at 7 o'clock, A. M., and we saw that all was useless. She gradually sunk, and died at 8 o'clock, 12 hours from the time I first saw her with Dr. A.

I would remark here, before closing this case, that the appearance of her countenance was such as to leave a strong impression upon my memory. It assumed the anxious expression which is pathognomonic of this dreadful disease. Her face was bedewed with a cold sweat, and in color it resembled, somewhat, sheet-lead, her eyes were sunken in their orbits, her fingers shriveled and cold, and in fact, the whole surface was cold and bedewed with a deathlike moisture.

*Case 4th.*—July 13th I was called to see Mr. Joseph D., æt. 36, residing in South street, below Sixth. He had a cold, corrugated skin, scarcely any pulse at the wrist, and an insatiable thirst, continually calling for cold water. He had had diarrhœa for four days previous to this attack.

Treatment: ℞.—Tinc. Opii. f. ʒss.

Tinc. Camph. gtts. xx.

Tinc. Capsicum, gtts. ʒss.

to be administered in brandy every half hour, during two hours. Two or three persons were constantly rubbing him with cayenne pepper and brandy, for at least one hour. Mustard was applied to the abdomen, hot pediluvia with mustard were scarcely felt by the patient, although his feet were kept in it 20 or 25 minntes. They were then taken out, wiped dry, and warm bricks applied, the body was then covered up closely with extra blankets, while warm drinks were diligently administered. In a short time profuse perspiration appeared, the pulse rose, and a general glow over the whole surface followed. At the next visit I found him much improved, having had a short nap. I called on the 14th, and found him still better, and directed quiniæ sulph., wine and water, &c. Discharged on the 4th.

*Case 5th.*—July 16th I entered the Moyamensing Hospital as resident Physician. The first admitted after entering on duty, was Elizabeth T. æt. 45, native of Ireland. She was attacked while watching over her daughter, who was, at this time, in a hopeless collapse. Mrs. T. was bled by Dr. N. about ℥ xij. half a drachm of calomel was given at one dose, followed up by tinct. opii., spt. camphor and hot cordial drinks; warm applications and frictions were employed. For the vomiting, creosote and sulph. morphiæ, in the usual dose, were given, with ice in the mouth, &c. Emp. canthar. 4 and 6 were applied to the epigastrium, but all to no purpose, as the vomiting continued until within a short time of her death. One thing it might be well to mention, she talked rationally, on sundry matters, until within a few minutes of her death; telling her friends that certain articles of wearing apparel were in pawn, and where they were to be found. When they were about to leave her, she extended her hand to them, inviting them to call again. In a few minutes afterwards she expired, making the time of her sickness 48 hours.

*Case 6th.*—Elizabeth M. æt. 27, (white). This was a robust woman, of strong constitution, who was admitted July 17th, at 10 A. M. f. ℥xvj. of blood were taken from the arm as soon as admitted, followed up by ℥ss. of calomel repeated in one hour; one-half dozen wet cups were applied over the epigastrium. She began to sink fast. A fly blister was then applied over the epigastrium, afterwards dressed with sulph. luinia. On the 18th we found her almost voiceless and pulseless. Carb. ammoniæ was given, five grains every half hour, when the pulse arose. She gradually improved, and continued doing so until the sixth day. Her friends were advised to take her home while she was still improving, but they did not do so. She then began to sink, of a low typhoid fever, and the evacuations from the bowels passed involuntarily for two days previous to her death. I would here remark that she was badly salivated, for which a gargle was made as follows:

℞.—Creosote f. ℥ss.
Flaxseed tea Oi.

to be used four or five times per day.

*Case 7th.*—Mrs. R., æt. 36, sister of case 4th, was attacked July 18th, with vomiting, purging, cramps in the abdomen and extremeties, coldness of the whole surface, and that shriveled appearance of the skin so peculiar to the cholera patient. Her eyes were sunken in their orbits, tongue cold; the surface bedewed with a deathlike clammy moisture. Treatment, opium grs. x. made into three pills, one to be taken every hour, with warm drinks frequently repeated. To relieve the vomiting, sulph. morphiæ was administered, and mustard to to the epigastrium, with warm applications externally. As soon as the vomiting ceased, carb. ammoniæ, five grains every half hour was given; the pulse soon rose, perspired freely, and from that moment she gradually improved. She was then put on tonics, such as quinia, wine and water, &c. In three or four days she was convalescent.

*Case 8th.*—Bridget G., æt. 39, native of Ireland, residing at the corner of 13th and Carpenter streets, was admitted into the Hospital July 23d. Her husband had died three days previous to her admission; with some four or five others that resided in the same house. There appeared, to me, to be a family in every room in the house, which was filthy in the extreme. She was brought to the Hospital, and admitted more as an act of humanity than otherwise, thinking it impossible to raise her; but contrary to all expectations, she recovered, and is now well. Treatment: opium in large doses with carb. ammo. v. grains every half hour, for twelve hours; warm drinks, gruel, &c., with the usual warm applications externally. On the 27th discharged *well.*

*Case* 9*th.*—Margaret G., daughter of the above, was admitted the same day. Discharged well on the 30th. Treatment same as her mother's.

*Case* 10*th.*—I was called July 25th, to see Mr. L., æt. 65, residing in 8th street, below Carpenter. He had been laboring under an affection of the bowels for some time, when he was taken with all the premonitory symptoms of Asiatic cholera. Treatment: opium in large doses, in combination with camphor. For the vomiting, creosote, sulph. morphiæ, with sinapisms to the epigastrium and ankles, with warm drinks, not forgetting the external applications, which are indispensibly necessary. On the 29th discharged *well.*

*Case* 11*th.*—Mary V., æt. 20, residing in Shippen Lane, below south, was brought to the Hospital July 28th. Her mother was in the last stage of collapse when Mary was brought in. So far gone was she, that her attending physician, Dr. W., thought it would be useless to bring her, and so it proved, as she died in a few hours after her daughter was taken to the Hospital. Mary recovered under the opium and sweating plan of treatment. Discharged on the 30th, well.

*Case* 12*th.*—Mr. J. W., æt. 35, native of Ireland, who resided at the corner of Willson's Court and Carpenter street, was admitted to the Hospital August 5th. His wife and daughter died a few days previous to his admission, of the same disease. His was truly a desperate case. I found him when brought in, in a profuse cold perspiration, no pulsation perceptible at the wrist, countenance and limbs shriveled and blue—in short all the symptoms of a fatal collapse. As usual, hot bricks, and bottles of hot water were applied along his sides, thighs, and to his feet, with extra blankets drawn closely around him. Two pills of five and a half grains of opium were given him in the space of half an hour. He fell into a deep sleep, perspired freely, and it was with great difficulty that he could be aroused; so badly was he narcotized that we rubbed his forehead with ice, and applied spts. ammoniæ to his nostrils; he then awoke, and with a smile so peculiar to him, expressed himself as "feeling better." Five grains of carb. ammo. were given him every half hour for 24 hours, with the usual drinks, toast water, brandy and water, warm gruel, &c. He was rubbed by the nurses with dry flannel several times, and was able to sit up and walk about the room in a few days. He was discharged convalescent on the 14th. In the language of Dr. Adkins, "his cure seemed to be almost miraculous."

In conclusion, sir, allow me to recapitulate the symptoms of epidemic cholera, as observed by me during the past summer.

1st. Purging or diarrhœa, the discharges generally of a dark, muddy appearance, in the majority of cases perfectly inodorous. Occasionally it had the appearance of "rice water evacuations."

2d. Vomiting, which was almost universal—the matter ejected having the appearance of "rice water," more so, certainly, than that from the rectum and bowels.

3d. Cramps, which were principally in the lower extremities.

4th. A peculiar husky voice which might be denominated the "cholera voice."

5th. The cyanosed hue and shrunken appearance of the face and hands, the latter appearing like those of a washerwoman, with blueness of the nails superadded.

6th. Sunken, hollow condition of the eyes and orbits.

7th. Small and corded pulse, in bad cases entirely absent from the wrist; this symptom, however, was not necessarily fatal, when accompanied by judicious treatment.

8th. An indisposition in the veins, and other tissues, to close, after incised or other wounds, the powers of the system seemed inadequate to the process of respiration.

9th. A thirst for cold water, which was quenchless.

10th. Coldness of the tongue and breath.

11th. The absence of the urinary secretion.

12th. Add to these the cold sweat, and adhesive, inelastic condition of the skin, with the mind at first clear, and sometimes even to the last moments, especially when the cases did not pass into the zyphora condition so common among the poorer patients.

In reference to the mode of treatment adopted in the above, and other cases, I must say that I give my unqualified adhesion to the opium and stimulating practice. As far as I have observed the treatment of cholera, I believe with Sutherland and his compeers, of the Royal College of Physicians, and of the Royal College of Surgeons of Edinburgh, as published in the September number of the "Medical Examiner," of this city, that "opium is the most powerful remedy, provided it be given in full and repeated doses within the first twelve hours at farthest, within the first 24 hours from the attack." The rationale of its action I do not pretend to give, but like Sydenham, with his laudanum, I am free to say, that it is much to be praised.

The patient should, by all means, be kept lying down, and not, if possible, be allowed to rise, even in bed, to stool.

The application of flannel and other rollers around the abdomen, in order to compress and sustain the viscera, was useful. The equalization of the temperature of the body was of the first importance, and the few cases of post mortem examinations with which I became acquainted, revealed little or no inflammation of the stomach, to account for the insatiable thirst, although these cases had had more or less stimulating medicines administered. Camphor, capsicum; and opium mixtures were generally found useful, with carbonate of ammonia, especially in the milder cases, but when the full force of the epidemic influence was felt upon the nervous centers, large doses of opium were the sheet anchor.

I am well aware that a large number of very respectable physicians have advocated a contrary course, viz: bleeding, calomel, dry friction, &c. My experience has taught me that the sooner the patient was raised from the dreadful depression, and digestion established, the sooner would the pulse rise, the skin becomes warm and elastic, the respiration regular ,and all the symptoms more favorable. No inflammation or organic disease appears ever to follow the retention of the blood in the veins, and adding as fast as possible to its amount by a judicious diet and moderately stimulating condiments and drinks. The chief thing, however, was to make the first grand impression upon the nervous, and consequently upon the muscular and all other systems, by means of a full dose of opium.

This accomplished, the treatment resolved itself into simply supporting the powers of life, and guiding the "vessel" through seas, somewhat rough it is true, but not dangerous, to a harbor of safety and comfort.

PHILADELPHIA, January, 1850.

